# The mitochondrial genome of the black-tailed dasyure (*Murexia melanurus*)

**DOI:** 10.1080/23802359.2019.1677526

**Published:** 2019-10-16

**Authors:** Ran Tian, Yuepan Geng, Patrick B. Thomas, Penny L. Jeffery, Thomas Y. Mutton, Lisa K. Chopin, Andrew M. Baker, Inge Seim

**Affiliations:** a Integrative Biology Laboratory, College of Life Sciences, Nanjing Normal University, Nanjing, Jiangsu, China;; b Ghrelin Research Group, Translational Research Institute-Institute of Health and Biomedical Innovation, School of Biomedical Sciences, Queensland University of Technology, Brisbane, Queensland, Australia;; c Australian Prostate Cancer Research Centre-Queensland, Translational Research Institute – Institute of Health and Biomedical Innovation, Queensland University of Technology, Brisbane, Queensland, Australia;; d Queensland Bladder Cancer Initiative, Translational Research Institute-Institute of Health and Biomedical Innovation, School of Biomedical Sciences, Queensland University of Technology, Woolloongabba, Queensland, Australia;; e School of Earth, Environmental and Biological Sciences, Queensland University of Technology, Brisbane, Queensland, Australia;; f Natural Environments Program, Queensland Museum, South Brisbane, Queensland, Australia;; g Comparative and Endocrine Biology Laboratory, Translational Research Institute-Institute of Health and Biomedical Innovation, School of Biomedical Sciences, Queensland University of Technology, Woolloongabba, Queensland, Australia

**Keywords:** Mitochondrial genome, marsupial, dasyure, Dasyuridae, New Guinea, *Murexia*

## Abstract

In this study, we report the mitochondrial genome of the black-tailed dasyure (*Murexia melanurus*) of New Guinea. The circular genome is 17,736 bp in length and has an AT content of 60.5%. Its gene content – 13 protein-coding genes (PCGs), 2 ribosomal (rRNA) genes, 21 transfer RNA (tRNA) genes, a tRNA pseudogene (*tRNA^Lys^*), and a non-coding control region (CR) – and gene arrangement are consistent with previous marsupial mitogenome assemblies.

The marsupial family Dasyuridae (or carnivorous marsupials) includes ∼75 species native to mainland Australia and Tasmania, New Guinea, and other adjacent islands (Baker and Dickman 2018). The dasyurid subfamily Phascogalinae comprises three genera: *Antechinus* and *Phascogale* of Australia, and *Murexia* of New Guinea. Although morphological similarities exist, the current evidence suggests these genera derived from a common ancestor some 12.5 million years ago (Mutton et al. 2019). *Murexia* spp. have not received as much attention in evolutionary studies as their Australian counterparts within Phascogalinae, and genetic information on the various constituent species within *Murexia* is poorly represented. Here, we describe the complete mitochondrial genome of the black-tailed dasyure (*Murexia melanurus*).

*Murexia melanurus* genomic DNA was extracted from ear tissue (voucher specimen ABTC46020) collected from Tibi, Papua New Guinea. Paired-end short-insert (200 bp) DNA libraries were sequenced by BGI (Hong Kong, China), to generate ∼30× genome coverage. Raw data were filtered using Flexbar v3.4.0 (Roehr et al. 2017). To remove microbial contaminants, we used bowtie2 v2.3.4.1 (Langmead and Salzberg 2012) to map reads to all bacterial and fungal sequences in NCBI Genomes, retaining 99.68% of reads. Two to 95 million reads were assembled using NOVOPlasty v2.7.2 (Dierckxsens et al. 2017), with the *ND1* coding sequence from a partial *M. melanurus* mitogenome (GenBank: KJ868127) (Mitchell et al. 2014) as a seed sequence and the parameters ‘Type = mito, K-mer = 39, Genome range = 16,000–22,000’. The subset with the lowest number of reads which yielded a circular genome was retained (here: 12 M reads; 0.26% were assembled into a 17,736 bp contig at 179× coverage). Geneious Prime v2019.1.3 (Biomatters Ltd., Auckland, New Zealand) was used to align 95 M reads against the assembled contig and generate a consensus genome sequence, with a 75% masking threshold. The genome was annotated using GenBank features of the northern quoll (*Dasyurus hallucatus;* accession no. NC_007630). Various genome features were compared to the Virginia opossum (*Didelphis virginiana*), a seminal species in the marsupial mitochondrial genetics literature [e.g. see Janke et al. (1994) and Nilsson (2009)].

The *M. melanurus* mitochondrial genome (GenBank: MK977600) is 17,736 bp and has a base composition of 32% A, 28.5% T, 14.1% G, and 25.4% C. As in other marsupials, the genome has 13 protein-coding genes (PSGs), 2 ribosomal (rRNA) genes, and 21 transfer RNA (tRNA) genes. The genome shares unique features with all marsupial mitogenomes reported to date. These include: an ‘ACWNY’ tRNA gene re-arrangement (Paabo et al. 1991); a *tRNA^Lys^* pseudogene (Janke et al. 1994; Dorner et al. 2001); and lack of an anticodon for aspartic acid, which is likely rescued by RNA-editing (Janke and Paabo 1993). In agreement with a recent molecular appraisal (Mitchell et al. 2014; Westerman et al. 2016), phylogenetic analysis revealed that *M. melanurus* and* Murexia habbema* are sisters, to the exclusion of the closely-related genera* Phascogale* and *Antechinus* (Figure 1). Maximum-likelihood (ML; estimated using IQ-TREE (Nguyen et al. 2015)) and Bayesian Interference (BI; implemented in MrBayes v3.2.7 (Ronquist and Huelsenbeck 2003)) gave the same tree topology.

**Figure 1.**
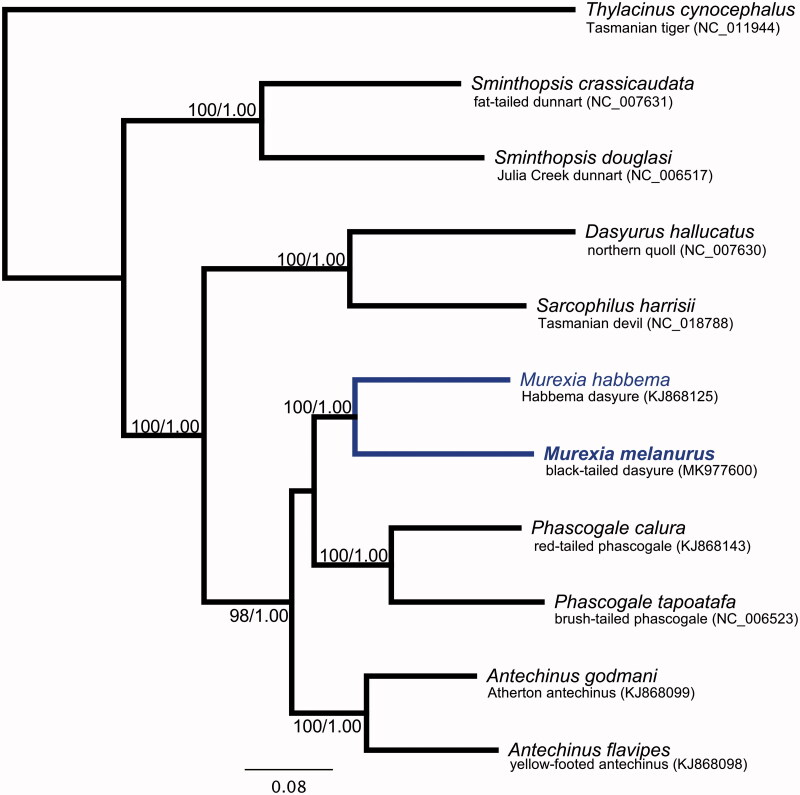
Phylogenetic tree of black-tailed dasyure (*Murexia melanurus*; indicated in bold blue font), nine other species in the marsupial family Dasyuridae, and the outgroup species *Thylacinus cynocephalus*. Because there are no complete mitogenomes from the genera *Antechinus* and *Murexia* in GenBank, phylogenetic reconstruction was performed with coding sequences of 12 protein-coding genes (excluding *ND6*). The number at each node is ML/BI bootstrap support value.

## Geolocation information

Geospatial coordinates for the black-tailed dasyure (*Murexia melanurus*) ear tissue collection: 6°11′S, 143°9′E
